# Trial-to-trial latency variability of somatosensory evoked potentials as a prognostic indicator for surgical management of cervical spondylotic myelopathy

**DOI:** 10.1186/s12984-015-0042-4

**Published:** 2015-05-29

**Authors:** Hongyan Cui, Yazhou Wang, Xiang Li, Xiaobo Xie, Shengpu Xu, Yong Hu

**Affiliations:** The Institute of Biomedical Engineering, Chinese Academy of Medical Sciences and Peking Union Medical College, No. 236 Baidi Road, Nankai District, 300192 Tianjin, China; Department of Orthopaedics and Traumatology, Li Ka Shing Faculty of Medicine, The University of Hong Kong, 12 Sandy Bay Road, Pokfulam, Hong Kong

**Keywords:** Cervical spondylotic myelopathy (CSM), Somatosensory evoked potentials (SEP), Prognosis, Single trial extraction, Second-order blind identification, Latency

## Abstract

**Background:**

Early detection of neural conductivity changes at the compressed spinal cord is important for predicting the surgical outcomes of patients with cervical spondylotic myelopathy (CSM). The prognostic value of median nerve somatosensory evoked potential (SEP) has been proposed previously. The present prospective study evaluates the use of trial-to-trial variability in SEP as a valuable predictor of neurological recovery after surgery of CSM.

**Methods:**

A total of 35 CSM patients who underwent surgery with up to 6-month follow-up were recruited in this study. SEP signals were recorded preoperatively. The single trial SEP was extracted by a newly developed second-order blind identification method. The postoperative recovery was assessed using the modified Japanese Orthopaedic Association. The correlation between the latency variability of trial-to-trial SEP and post-operative recovery ratio was analyzed. The prognostic value of trial-to-trial SEP for CSM was evaluated using a receiver operator characteristic curve which can accurately reflect the relationship between sensitivity and specificity of a diagnostic method and represent the accuracy of prognosis.

**Results:**

The correlation coefficient of trial-to-trial latency variability and the 6-month recovery ratio was statistically significant (r = −0.82, *P* < 0.01). The trial-to-trial SEP had a higher prognostic accuracy (AUC = 0.928, *P* < 0.001) with an optimal prognostic value of 9.25 % compared with averaged SEP when the threshold of recovery ratio was 40 %, and was more sensitive (93.80 %) than the averaged SEP (43.80 %).

**Conclusions:**

These findings indicate that the latency variability of trial-to-trial SEP reflect the recovery ratio of CSM patients after surgery. It is suggested that the latency variability of trial-to-trial SEP is useful for predicting the surgical outcomes for patients with CSM, which would be a potential indication of surgical treatment for CSM to help decision making of surgical planning for CSM patients.

**Electronic supplementary material:**

The online version of this article (doi:10.1186/s12984-015-0042-4) contains supplementary material, which is available to authorized users.

## Background

Cervical spondylotic myelopathy (CSM) is the most common cause of spinal cord disorder among the elderly over 55 years old [1, 2]. Surgical decompression is considered as the most effective treatment for patients with CSM [3]. However, the accurate prognosis of surgical outcome is still a problem during clinical treatment decision making [3–5]. Postoperative outcomes are affected by multiple prognostic factors [6], making the clinical outcome of CSM unexpectedly complex, and generates variability in the prognosis.

Several studies have investigated the prognostic value of somatosensory evoked potential (SEP) for CSM [7–10]. One previous study demonstrated that preoperative somatosensory evoked potentials can provide important information correlated with prognosis [11], and preoperative SEP showed good correlation with CSM disability [10, 12].

The latency and amplitude of SEP are two sensitive parameters reflecting nerve conductivity along the spinal cord. The abnormal latency by itself is a better predictor of outcome than is abnormal amplitude by itself [10], because changes in latency are more sensitive to spinal cord injury and neurological recovery [8, 13, 14]. Previous studies employed across-trial ensemble averaging method of 100 to 500 trials to obtain a measurable SEP signal [10], since SEP signals are usually accompanied by noise from movement artifact, other electrophysiological signals, and environmental electromagnetic activity [15, 16]. A previous paper reported that averaging SEP cannot detect the time-variant neurological abnormalities [13], which is supposed to be a main feature in CSM. The pathological progress of CSM includes demyelination, axon loss and neuronal apoptosis [17], which are incomplete damage to the spinal cord. The pathological changes in CSM lead to the altered or disturbed connection induced by the alterations to the neural signals and sources, representing abnormalities of conduction velocity and dispersion of conduction due to electrophysiological derangement [18]. With the progress of myelopathy, the increasing amount of demyelination and axon loss would lead large variation in single trial SEP latency. Because the extent of pathological changes is closely correlated with the prognosis, trial-to-trial latency variability would be a useful measurement to indicate the prognosis of CSM treatment. For this reason, it is worthy to evaluate the usefulness of cross-trial dynamics of SEP in CSM prognosis.

However, use of ensemble averaging method with a large number of SEP trials may minimize time-variant abnormalities of conductivity [15]. To obtain a reliable estimation of single evoked potential response, several methods have been developed under poor signal-to-noise ratio [13, 19--21]. In 1979, Kearney proposed to use Weiner filtering to the estimate tonic electromyography activity responses after electrical stimulation of the foot [22]. Results showed a significant reduction in the number of averaging, but it did not prove the usefulness in single trial SEP extraction. It is much more difficult to extract single-trial SEP than other evoked potentials like evoked electromyography, visual evoked potentials and audio evoked potentials. Recently, improved performance has been reported for blind source separation, which is a technique that recovers unknown source signals from mixed and observed data sets [13, 19, 20]. Compared with other blind identification algorithms for SEP detection, second-order blind identification has many advantages such as simplicity, reliability, robustness, and applicable Gaussian signals [13, 19, 20]. More importantly, second-order blind identification is robust for short serial-signals [23]. Therefore, second-order blind identification offers a favorable alternative for detecting neural transmission variation [13].

Considering the usefulness of SEP latency variation in detecting neurophysiological dysfunction [13], the aim of this study is to evaluate the prognostic value of latency variability of trial-to-trial SEP in neurological recovery after surgery of CSM.

## Methods

### Subjects

A total of 35 patients with CSM (19 men and 16 women), who underwent cervical surgery between April 2010 and September 2012 were recruited. The mean age was 60.4 ± 11.2 years (range, 55–70 years) and the mean duration of symptoms was 43 ± 51 weeks (range, 24–560 weeks). All patients were followed up postoperatively for 6 months. These patients had no other neurologic diseases. All patients provided informed consent, and the procedures were approved by the Institutional Review Board of the University of Hong Kong/Hospital Authority Hong Kong West Cluster.

### Clinical assessment

To evaluate the clinical outcome for surgical management of CSM, the Japanese Orthopaedic Association (JOA) score was used to assess the pre- and postoperative clinical condition [10]. The JOA score is a useful tool to evaluate the severity of neurologic deficits. The maximum JOA score is 17, including motor dysfunction of the upper (4 scores) and lower extremities (4 scores), sensory deficits of the limbs (4 scores) and trunk (2 scores), and sphincter dysfunction (3 scores) [24]. Low score represents the severity of neurological deficit [10, 25]. All patients were clinically evaluated by the JOA score before the operation and at 6 months postoperatively. Surgical outcome with 6 months follow-up was evaluated by the recovery ratio and calculated as follows [10, 25]:

$$ \mathrm{Recovery}\kern0.5em \mathrm{ratio}=\frac{\mathrm{postoperative}\kern0.5em \mathrm{J}\mathrm{O}\mathrm{A}\kern0.5em \mathrm{score}\hbox{-} \mathrm{preoperative}\kern0.5em \mathrm{J}\mathrm{O}\mathrm{A}\kern0.5em \mathrm{score}}{17\kern0.5em \mathrm{points}\hbox{-} \mathrm{preoperative}\kern0.5em \mathrm{J}\mathrm{O}\mathrm{A}\kern0.5em \mathrm{score}}\times 100\% $$

while the 17 points is the maximal scale of JOA.

### Somatosensory evoked potential recording

Median nerve SEP were recorded preoperatively to determine diagnosis and prognosis of CSM. During the SEP test, the patient was asked to lie on a couch in a warm and semi darkened room.

The median nerve at the wrist was stimulated by constant current stimulus ranging 10 to 30 mA, with a frequency between 5.1 and 5.7 Hz and duration of 0.3 ms. The SEP signals were recorded at Cz´ (2 cm posterior to Cz, 10–20 international system of EEG electrode placement), C3 and C4 (10–20 international system of EEG electrode placement) and Cv (on the cervical spine over the C2 process) with the reference electrode at Fz (10–20 international system of EEG electrode placement). All signals were recorded with a sampling rate of 5 kHz for each recording channel by an evoked potential recording system (YRKJ-A2004; Zhuhai yiruikeji Co., Ltd., Zhu Hai, China), using 20–2000 Hz bandpass filter and automatic artifact rejection performed. The automatic artifact rejection was designed to reject the bad trials with large amplitude after 5 ms of sweep. A continue SEP of 100 trials was recorded at left and right median nerve respectively, saved as 2 sets of single trial SEP data for further processing.

### Somatosensory evoked potential processing

Single trial SEP was determined by second order blind identification with a reference algorithm [19], which is developed from the traditional blind source separation algorithm [21]. In the classical second order blind identification, a rotation matrix *V*is chose to jointly diagonalize all of them by minimizing,

1 $$ {{{\displaystyle {\sum}_{\tau }{\displaystyle {\sum}_{i\ne j}\left({V}^T{R}_{\tau }V\right)}}}_{ij}}^2 $$

the sum of the squares of the off-diagonal entries of the matrix products *V*^*T*^*R*_*τ*_*V*, via an iterative process. *R*_*τ*_ denotes a set of time-lagged covariance matrices defined as

2 $$ {R}_{\tau }=E\left(x\left(t+\tau \right)x{(t)}^T\right),\tau \ne 0 $$

Users can set a threshold parameter for the angle of rotation of *V*. When the angle is smaller than the threshold, the iterative process ends. The final estimate of the unmixing matrix is:

3 $$ W=VB $$

which is used to derive the separated components.

**Fig. 1:**
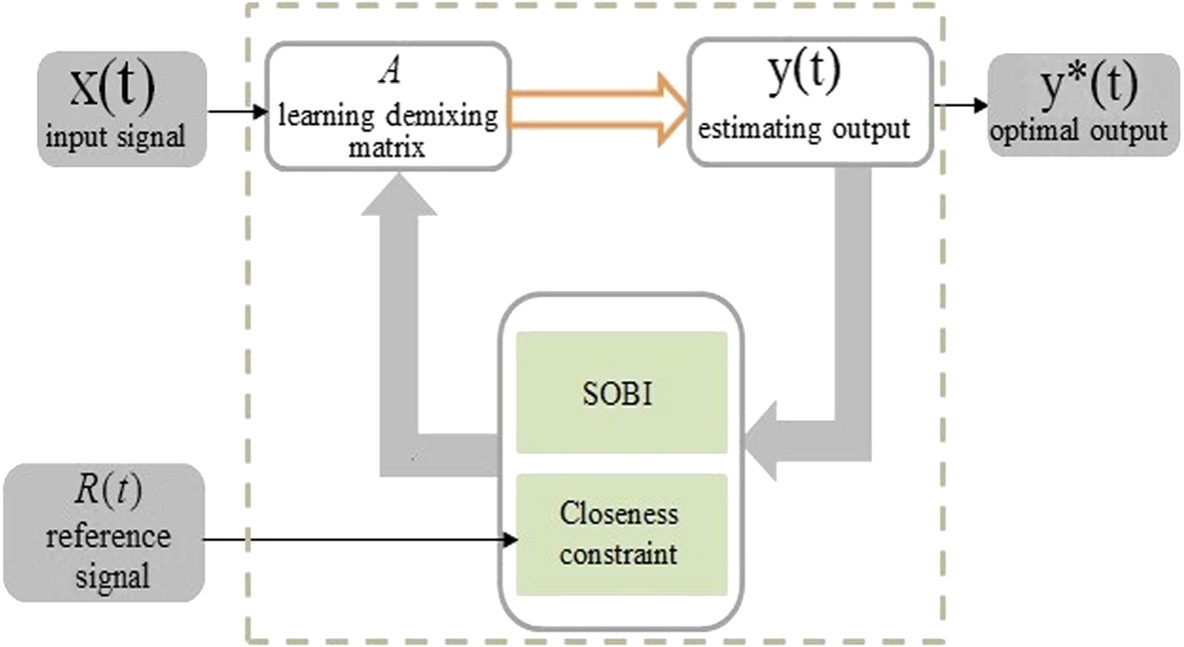
Block diagram of second order blind identification with a reference algorithm to analyze single trial SEP. The framework is presented with input signal, reference signal, output signal and the main processing part of the second order blind identification with a reference algorithm. When the input signal is *X* (*t*), the learning demixing matrix *A* must be adjusted by both second order blind identification and the closeness constraint relative to the output *y* (*t*) according to reference signals *R* (*t*), Until *y* (*t*) = *y* ^*^is the constraint condition of closeness between the estimated output *y* (*t*) and the reference *R* (*t*), the optimal output *y*(*t*) = y* can be obtained

As shown in Fig. 1, the recorded multi-channel SEP signals *X* (*t*) = [*x*_1_, *x*_2_, …, *x*_*M*_] ^*T*^ are assumed to be a mixture of source components where *A* is a *M* × *N* unknown full rank mixing matrix, with *M* ≥ *N. y* (*t*) is an estimated output, and *R* (*t*) is the reference signal.

4 $$ X(t)={A}^TS(t) $$

The second order blind identification with a reference is designed to extract a single desired source and discard the rests which are irrelevant to the reference signal. In this study, only the main component in post-tibial nerve short-latency SEP is of interest, and then the computational load can be greatly reduced. In this situation, the goal of this algorithm was to identify a demixing vector *w* (one column of the learning demixing matrix *W*) such that the output signal is equal to the desired source signal *S* (*t*):

5 $$ y(t)={w}^TX(t)={w}^T{A}^TS(t)=S(t) $$

Where *S* (*t*) = [*s*_1_, *s*_2_, …, *s*_*N*_] is a mixtures of *N* independent source signal.

Different from the classical second order blind identification, second order blind identification with a reference needs another objective function to establish the relationship between *y* (*t*) and both equation (1) and *R* (*t*). The first part contrast function is to minimize *J* (*y*) defined as

6 $$ J(y)=-{{\displaystyle {\sum}_{\tau }E\left(y(t)y{\left(t-\tau \right)}^T\right)}}^2 $$

The closeness between the estimated output *y* and the corresponding reference *r* is measured by *ɛ* (*y*, *r*), whose minimum value indicates the optimal output y^*^ of *J* (*y*). A threshold *ξ* can be used as a constraint condition of closeness such that

7 $$ g(y)=\varepsilon \left(y,r\right)-\xi \le 0 $$

is satisfied only when *y* = y^*^. By incorporating (6) with (7), second order blind identification with a reference can be formulated as follows:

minimize *J*(*y*) = − ∑_*τ*_*E*(*y*(*t*)*y*(*t* − *τ*)^*T*^)^2^

subject to *g* (*y*) ≤ 0 and *h* (*y*) = 0 (8)

where *h*(*y*) = *E*(*yy*^T^) − 1 is included to restrict the output have unit variance.

By adopting the Lagrange multipliers method for optimal solution, the augmented Lagrangian function is given as

9 $$ \begin{array}{r}L\left(w,\mu, \lambda, z\right)=J(y)+\mu \widehat{g}(y)+\frac{1}{2}\gamma {\left\Vert \widehat{g}(y)\right\Vert}^2\\ {}+\lambda h(y)+\frac{1}{2}\gamma {\left\Vert h(y)\right\Vert}^2\end{array} $$

where *μ* and *λ* are Lagrange multipliers for the inequality constraint and the equality constraint respectively, and *γ* is a scalar penalty. *z* is a slack variable to convert the inequality constraint into equality constraint.

The minimization of (9) with respect to *z* can be performed explicitly for fixed *w* as follows:

10 $$ {\displaystyle \underset{z}{ \min }}L\left(\mathbf{w},\mu, \lambda, z\right)={\displaystyle \underset{z^2\ge 0}{ \min }}\left\{\mu \left(g(y)+{z}^2\right)+\frac{1}{2}\gamma {\left\Vert g(y)+{z}^2\right\Vert}^2\right\} $$

A Newton-like learning algorithm is used to find the optimal value as [19]

11 $$ \varDelta w=-\eta {\left(\frac{\partial^2L\left(w,\mu, \lambda \right)}{\partial {w}^2}\right)}^{-1}\frac{\partial L\left(w,\mu, \lambda \right)}{\partial w} $$

where *η* is the learning rate. The Lagrange multipliers μ and *λ* are updated as

12 $$ \varDelta \mu = \max \left\{-\mu, \gamma g(y)\right\} $$

13 $$ \varDelta \lambda =\gamma h(y) $$

Until the error, |*J* (*y*) _*k*+1_ − *J* (*y*) _*k*_| is small enough, otherwise go back to update the vector *w* by *w*_*k* + 1_ = *w*_*k*_ + *Δw*. Details of the second order blind identification algorithm is seen in Additional file 1.

After being processed by the second order blind identification algorithm, recordings were visually analyzed for the presence of the main peaks N1-P1, and the measured parameters of cortical response included peak latency of N1. Single trial SEP were analyzed in 100 SEP recordings. After the standard deviation of latency was calculated from results of the 100 recordings, the latency variability of trial-to-trial SEP was defined as the ratio between the standard deviation and the mean value, which was calculated as follows:

$$ \mathrm{Latency}\ \mathrm{variability}\kern0.5em =\frac{\mathrm{standard}\ \mathrm{deviation}\ }{\mathrm{mean}\ \mathrm{value}} \times 100\% $$

In each subject, latency variability of trial-to-trial SEP was calculated in left and right median nerve SEP. In these two values of latency variability, the lowest variability was selected for this subject for prognosis evaluation.

### Statistical analysis

Data were presented as the mean ± standard deviation. The correlation between the trial-to-trial SEP variability and the surgical outcome was determined using the Pearson correlation coefficient analyzed with the software program SPSS 16.0. Statistical significance was designated at α = 0.05 using a bilateral test (two-tailed). A P-value < 0.05 and an absolute correlation coefficient ≥ 0.50 were considered a significant linear correlation.

In addition, the receiver operator characteristic curve was used to evaluate the performance of second order blind identification with a reference algorithm in predicting the prognosis of CSM. Receiver operator characteristic curve can accurately reflect the relationship between sensitivity and specificity of a diagnostic method compared with a standard method and can represent the diagnostic accuracy of the method. The area under the curve (AUC) of receiver operator characteristic is between 1.0 and 0.5. Assuming AUC > 0.5 [26], a higher AUC indicates better diagnostic value of the method as follows: AUC = 0.5–0.7 indicates low accuracy; AUC = 0.7–0.9 indicates certain accuracy; and an AUC > 0.9 indicates high accuracy [26]. An AUC = 0.5 indicates no diagnostic value; that is, the diagnostic method does not work. In this study, JOA score is defined as the standard method and the variability of trial-to-trial SEP and averaged SEP were compared as diagnostic methods.

## Results

**Fig. 2:**
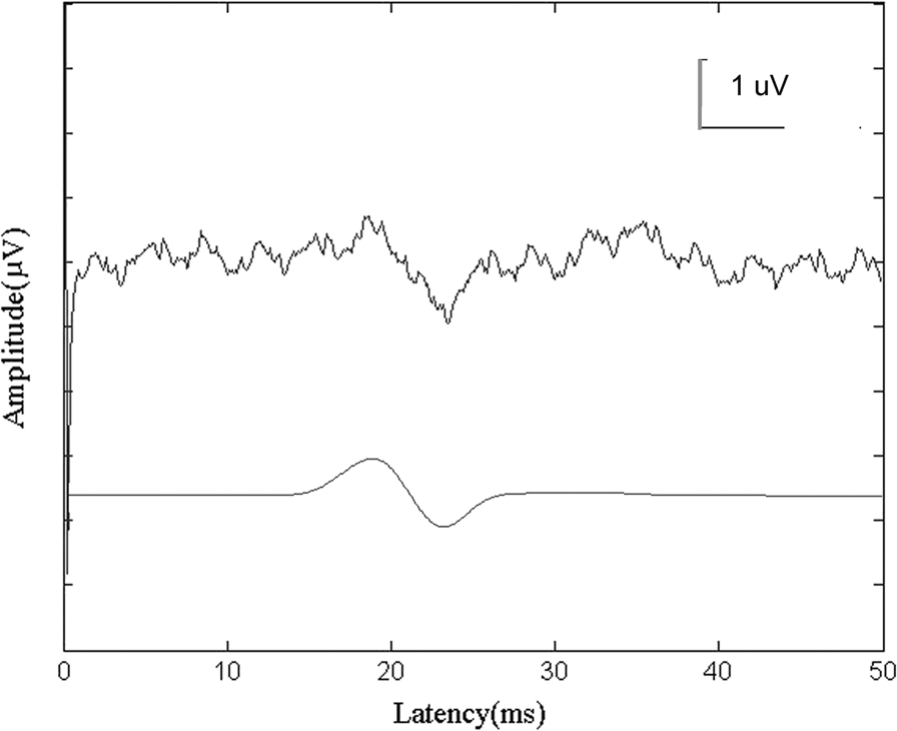
Representative ensemble averaging waveform generated from 50 raw SEP and a sample of single-trial SEP. The SEP waveforms are compared that extracted by ensemble averaging method and second order blind identification with a reference algorithm. The above signal is the ensemble averaging waveform, and the below is the single-trial SEP. They have similar shape within 15 ms and 25 ms

**Fig. 3:**
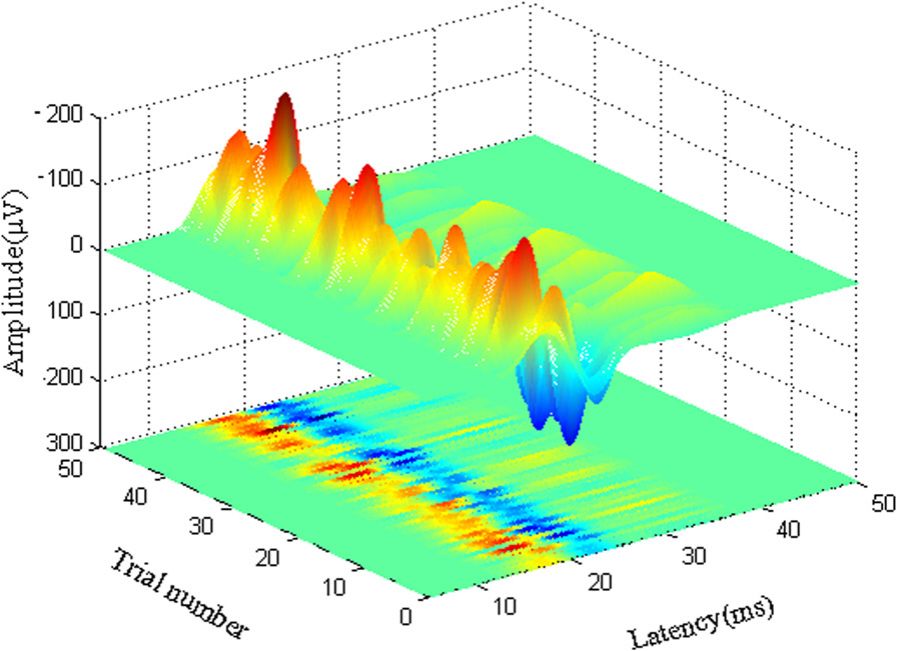
Single trial SEP measured using second order blind identification with a reference in CSM patients. The 50 single trial SEP are presented with latency and amplitude values. The above is the three dimensional map of single-trial SEP in latency-amplitude-trial number, and the below is the projection on latency-trial number coordinate. The color presents the values of single-trial SEP amplitude with a latency between 10 ms to 20 ms

The latency in all 35 patients determined by the ensemble averaging method was 19.25 ± 1.90 ms in mean ± standard deviation, and the latency variability determined by second order blind identification with a reference method was 9.55 ± 2.08 ms. Figure 2 shows a representative ensemble averaging waveform measuring 50 raw SEP in one CSM patient and a sample of single-trial SEP. Extracted by second order blind identification with a reference, a series of single trial SEP have different latency values, which demonstrates the latency variability of trial-to-trial SEP (Fig. 3).

**Fig. 4:**
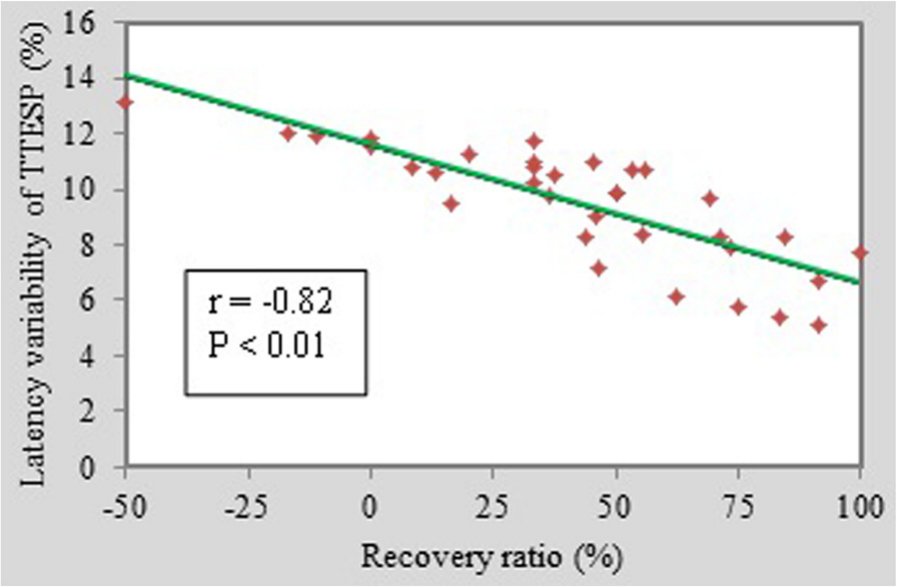
Correlation between the latency variability of TTSEP and the 6-month postoperative recovery ratio. The latency variability of TTSEP and the 6-month postoperative recovery ratio in percentage are compared using correlation analysis. The red stars are the scattered recovery ratio and latency variation of TTSEP of 35 patients distributing regularly, and the green line is the trend line between them. The statistic correlation coefficient r is −0.82 presenting a significant negative correlation (*P* < 0.01)

The preoperative JOA score increased from 4 to 14 with a mean value 10.68, and the postoperative JOA score increased from 7.5 to 17 with a mean value 13.90. The mean recovery ratio was 41.08 % (ranged from 0 to 100 %). Figure 4 shows the correlation between the latency variability of the trial-to-trial SEP and the recovery ratio. The data points were regularly distributed in the Cartesian coordinate plane, and the trend line showed a strong correlation (r = −0.82, *P* < 0.01), which indicated that latency variability of the trial-to-trial SEP was correlated to postoperative outcome.

**Fig. 5:**
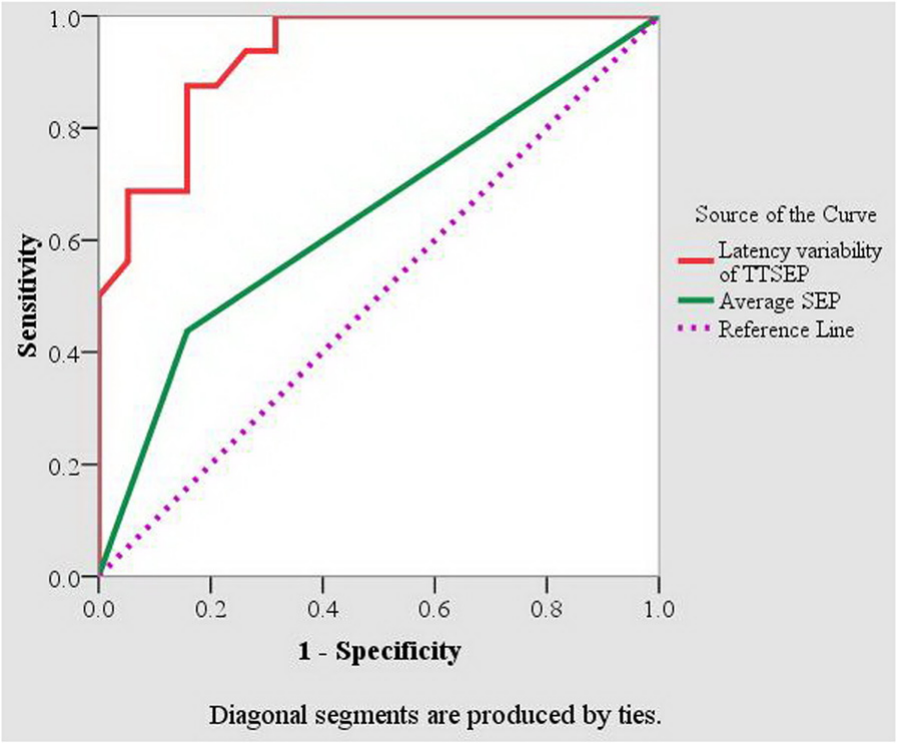
Receiver operator characteristic curves for the latency variability of TTSEP and averaged SEP at a 40 % recovery ratio threshold. The diagnostic methods of variability of TTSEP and averaged SEP were compared according to the recovery ratio with their specificity and sensitivity. Red line is presented latency variability of TTSEP, green line is averaged SEP, and pink dashed line is reference line considered the diagnostic criteria with a threshold 40 %. Bigger area under the curve (AUC) of red line indicates better diagnostic value of the variability of TTSEP method for CSM than averaged SEP

According previous report [27], the recovery ratio of 40 % was considered the diagnostic threshold criteria for an acceptable good prognosis. The optimal diagnostic threshold value of latency variability of trial-to-trial SEP was 9.25 %. When the preoperative latency variability of trial-to-trial SEP was greater than 9.25 %, the recovery ratio was less than 40 %, and when the preoperative latency variability of trial-to-trial SEP was less than 9.25 %, the recovery ratio was greater than 40 %. In averaging SEP, the diagnostic threshold criterion was defined as delayed latency (18.41 + 2.5*0.71 = 20.19 ms) [10]. In this group of patients, the sensitivity of prognosis by trial-to-trial SEP presented 93.80 %, while the sensitivity by averaging SEP was 43.80 %. Figure 5 illustrates the receiver operator characteristic curve with a 40 % recovery ratio as the criterion of good prognosis. The AUC of latency variability of trial-to-trial SEP was 0.928 (*P* < 0.001), and the AUC of averaged SEP was 0.640 (*P* < 0.001), indicating that the latency variability of trial-to-trial SEP had a higher accuracy for CSM prognosis.

## Discussion

This study demonstrates that latency variability of trial-to-trial SEP is closely correlated with recovery ratio after surgical management of CSM. In comparison with conventional averaging SEP, the latency variability of trial-to-trial SEP presented much higher sensitivity and specificity in predicting surgical outcomes of CSM. This finding suggests that latency variability of trial-to-trial SEP is able to provide a precise prognosis of CSM surgery.

The prognosis for neurologic recovery after surgical treatment of CSM is important in informing both patients and surgeons of what to expect [28, 29]. The pathological progress of CSM includes demyelination, axon loss and neuronal apoptosis [17], which change the conductivity of spinal cord. The diagnostic value of SEP in CSM patients has been reported [7–10, 12], and some studies have suggested that SEP may be useful for predicting the postoperative prognosis in CSM patients [11]. The prognostic value of SEP abnormalities in CSM patients has not been systematically studied, particularly the correlation between SEP variability and the postoperative outcomes in CSM patients. SEP can reflect the nervous status along a particular pathway in response to an external stimulus [7]. However, in clinical conditions, SEP signals are usually flooded by numerous noise signals from the patient and the environment [15, 20], which makes detection of the SEP peak difficult and often results in inaccurate measurements. Ensemble averaging is often used to enhance the signal-to-noise ratio, but it does not allow evaluation of time-varying associated features that may be more suitable for dynamic variability analysis [13, 30]. Early detection of neural conductivity changes at the compressed spinal cord would be an indication for surgical decision of patients with CSM.

Bouchard et al. reported that intraoperative SEP changes are associated with the short-term recovery ratio of CSM, but there is no obvious correlation with the long-term recovery ratio, which decreases its prognostic value [12]. In this study, we analyzed the neurological recovery ratio at 6 months postoperatively. The results showed a significant correlation between the preoperative trial-to-trial SEP variability and the postoperative clinical improvement measured by the recovery ratio, which represents the neurological recovery of CSM patients. This finding is consistent with that reported by Matsukado [31] and Hu [10].

Analysis of single trial signals, rather than the across-trial ensemble averaging signals, can detect changes in SEP [13], and many investigators have described single trial signal processing algorithms to improve the evoked potential signal and better understand the waveform changes over time [19, 32–35]. The traditional second order blind identification processed signal requires comparatively less time averaging [19]. Constrained second-order blind identification is a blind source separation technique based on two orders of blind source separation to remove the irrelevant signal components [23], extract the signal components associated with the reference algorithm, and isolate the desired signal, which greatly reduces the amount of computation [19]. Recently, second order blind identification has been confirmed as one of the best techniques for single trial SEP detection [19]. In addition, one previous study found that latency measurements are a more reproducible and reliable indicator of neurologic deficits, and latency is a better predictor of clinical outcome [10]. Therefore, in this study, we calculated the latency variability of trial-to-trial SEP to evaluate the utility of the SEP for predicting the prognosis of CSM patients. To clarify the correlation between the trial-to-trial SEP latency variability and postoperative recovery in CSM patients, we systemically analyzed the relationship between the latency variability of trial-to-trial SEP and the recovery ratio of CSM. In addition, the receiver operator characteristic was used to evaluate the prognostic value of latency variability of trial-to-trial SEP. The results showed that when using the single trial SEP extracted by second order blind identification with a reference, it was not only easier to identify the peak, but also to obtain the variability in trial-to-trial SEP. As seen in Fig. 3, the latencies change between the various trials was apparent.

In the present study, the receiver operator characteristic curve indicated that the latency variability of trial-to-trial SEP had a much better precision in prognostic value for CSM (AUC = 0.928, *P* < 0.001) than did averaged SEP (AUC = 0.640, *P* < 0.001). In a previous study [25], the precision of prognosis of preoperative JOA score was evaluated by ROC curve as AUC = 0.489, which suggested the merit of trial-to-trial SEP as a good predicting factor for prognostics evaluation of CSM.

## Conclusions

Second order blind identification with a reference can detect changes in latency of trial-to-trial SEP in patients with CSM. Furthermore, the variability of the trial-to-trial SEP signal was significantly correlated with the neurological recovery ratio measured by the JOA score and was more sensitive than the averaged SEP. Therefore, preoperative variability of trial-to-trial SEP may be more suitable for measuring changes in neurological function of CSM patients and is likely a better predictor of postoperative prognosis for CSM patients. Trial-to-trial SEP may be used to predict the magnitude of clinical improvement in patients undergoing surgery for CSM treatment, which is useful for surgical and prognostic planning for CSM.

## Additional file

Additional file 1:
**Single trial SEP extraction algorithm using second order blind identification with a reference.**

## Abbreviations

CSM: Cervical spondylotic myelopathy
SEP: Somatosensory evoked potential
TTSEP: Trial-to-trial somatosensory evoked potential
JOA: Japanese orthopaedic association scoring system
AUC: Area under the curve
