# Supplementary material for: Trial-to-trial latency variability of somatosensory evoked potentials as a prognostic indicator for surgical management of cervical spondylotic myelopathy
Source: J Neuroeng Rehabil. 2015 May 29;12:49. doi: 10.1186/s12984-015-0042-4 (PMC4467682; doi:10.1186/s12984-015-0042-4)
Supplement: Additional file 1: — Single trial SEP extraction algorithm using second order blind identification with a reference. [file 12984_2015_42_MOESM1_ESM.docx]

**Single trial SEP extraction algorithm using second order blind identification with a reference**

**(1) Second-order blind identification algorithm**

Suppose SEP signals are composed of a mixture of source components $S(t)$ as follows:

$X(t)=A^{T}S(t)$ (1)

where $X(t)=[x_{1},x_{2},...,x_{M}]$, $S(t)=[s_{1},s_{2},...,s_{N}]$ and $A$ is a $M\times N$unknown full rank mixing matrix, with $M\geq N$. The blind source separation algorithm is to estimate the unknown $A$ and $S(t)$.

To estimate matrix$A$, the blind source separation is to determine an $M\times N$ demixing matrix $W$, such that the output signal $y(t)$ is equal to the desired source signal $S(t)$:

$y(t)=W^{T}X(t)=w^{T}A^{T}S(t)=S(t)$ (2)

The classical second-order blind identification algorithm proceeds in two stages to find the solution. First, the observed signals $X(t)$ are zero-meaned as follows:

$\hat{X}(t)=B(X(t)-E(X(t)))$ (3)

where $E\left(\cdot\right)$is an average of $X(t)$, and the matrix $B$ is an identity matrix by:

$\begin{matrix} B=\Lambda^{-\frac{1}{2}}U^{T} \\ \mathop{}\limits\mathop{=}\limits diag(\lambda_{l}^{-\frac{1}{2}})U^{T} \end{matrix}$ (4)

where $U$is the principal component analysis components of $X(t)$.

Secondly, a delay of $\tau$ is set to compute correlation matrices $R_{\tau}$ between $\hat{X}(t)$and its temporally shifted version:

$R_{\tau}=sym(E(\hat{X}(t)\cdot\hat{X}(t-\tau))^{T})$ (5)

where $sym(.)$ is an asymmetric matrix.

After calculating $R_{\tau}$, a rotation matrix $V$ is chosen to jointly diagonalize $R_{\tau}$ via an iterative process by minimizing the sum of $VR_{\tau}V^{T}$ as follows:

min $\sum_{\tau} \sum_{i\neq j} (VR_{\tau}V^{T})^{2}$ (6)

which is an iterative process to make the angle of rotation $V$to a setting threshold.

When $V$becomes lower than the setting threshold, the process terminates to develop the demixing matrix $W$as

$W=A^{-1}=VB$ (7)

**(2) Second-order blind identification with reference**

In second-order blind identification algorithm with reference, the demixing matrix $W$ must be modified by second-order blind identification as well as the constraint condition to the output $y(t)$. In particular, the optimization problem of second-order blind identification algorithm with a constraint is to transform minimizing function (6) to function expressed as:

min $F(R_{\tau},V)=\sum_{\tau} \sum_{i\neq j} \left|VR_{\tau}V^{T}\right|^{2}$ (8)

According to equation (5) and (7), it follows that:

$\begin{matrix} F(R_{\tau},V)=\sum_{\tau} \sum_{i\neq j} \left|VE(\hat{X}(t)\hat{X}(t)^{T})\right|^{2} \\ \mathop{}\limits\mathop{}\limits\mathop{}\limits\mathop{}\limits\mathop{}\limits\mathop{}\limits=\sum_{\tau} \sum_{i\neq j} \left|VE(BX(t)X(t)^{T}B^{T})V{}^{T}\right|^{2} \\ \mathop{}\limits\mathop{}\limits\mathop{}\limits\mathop{}\limits\mathop{}\limits\mathop{}\limits=\sum_{\tau} \sum_{i\neq j} \left|VBE(X(t)X(t)^{T})B^{T}V{}^{T}\right|^{2} \\ \mathop{}\limits\mathop{}\limits\mathop{}\limits\mathop{}\limits\mathop{}\limits\mathop{}\limits=\sum_{\tau} \sum_{i\neq j} \left|WE(X(t)X(t)^{T})W{}^{T}\right|^{2} \end{matrix}$ (9)

The contrast function can be expressed as:

$\begin{matrix} J(y)=-\sum_{\tau=1}^{p} (wE(X(t)X(t)^{T})w{}^{T})^{2} \\ \mathop{}\limits\mathop{}\limits\mathop{}\limits=-\sum_{\tau=1}^{p} (E(wX(t)X(t-\tau)^{T}w{}^{T}))^{2} \\ \mathop{}\limits\mathop{}\limits\mathop{}\limits=-\sum_{\tau=1}^{p} (E(y(t)y(t-\tau){}^{T}))^{2} \end{matrix}$ (10)

The closeness between the estimated output $y$and the corresponding reference $r$ is measured by$\varepsilon(y,r)=E\left\{(y-r)^{2}\right\}$. A threshold $\xi$ is set to constrain the process such that

$g(y)=\varepsilon(y,r)-\xi\leq0$ (11)

is satisfied only when $y=y^{*}$. By incorporating (10) with (11), second order blind identification with a reference for SEPs extraction can be formulated as follows:

min $J(y)=-\sum_{\tau} E\left(y(t)y(t-\tau)^{T}\right)^{2}$

subject to $g(y)\leq0$ and $h(y)=0$ (12)

where $h(y)=E\left(yy^{\text{T}}\right)-1$ is included to restrict the output have unit variance.

Making an equality constraint, a slack variable z is introduced, i.e., $\hat{g}(y)=g(y)+z^{2}=0$. By adopting the Lagrange multipliers method for optimal solution, the augmented Lagrangian function is given as:

$\begin{matrix} L(w,\mu,\lambda,z)=J(y)+\mu\hat{g}(y)+\frac{1}{2}\gamma\left\|\hat{g}(y)\right\| ^{2} \\ +\lambda h(y)+\frac{1}{2}\gamma\left\|h(y)\right\| ^{2} \end{matrix}$ (13) where $\mu$ and $\lambda$ are Lagrange multipliers for the inequality constraint and the equality constraint respectively, and $\gamma$ is a scalar penalty, $z$ is a slack variable, and denotes the Euclidean norm.

Replacing $\hat{g}(y)$ in (13) with $g(y)$ and $z$, the minimization of (13) with respect to $z$ can be performed explicitly for fixed $w$ as follows:

$\text{min}_{z} \mathop{}\limits L(w\text{,}\mu\text{,}\lambda\text{,}z)=\text{min}_{z^{2}\geq0} \left\{\mu(g(y)+z^{2})+\frac{1}{2}\gamma\left\|g(y)+z^{2}\right\|^{2}\right\}$ (14)

where $\frac{\partial L(w,\mu,\lambda,z)}{\partial z}=0$, the optimal value $z^{*}$of $z$ satisfies the following relationship:

$(z^{*})^{2}=max\left\{0,-(\frac{\mu}{\gamma}+g(y))\right\}$ (15)

which yields

$\text{min}_{z} \mathop{}\limits L(w\text{,}\mu\text{,}\lambda\text{,}z)=\left\{\begin{matrix} -\frac{1}{2}\frac{\mu^{2}}{\gamma}\mathop{,}\limits\mathop{}\limits\mathop{}\limits\mathop{}\limits\mathop{}\limits\mathop{}\limits\mathop{}\limits\mathop{}\limits\mathop{}\limits\mathop{}\limits\mathop{}\limits\mathop{}\limits\mathop{}\limits\mathop{}\limits\mathop{}\limits\mathop{}\limits\mathop{}\limits\mathop{}\limits\mathop{}\limits-(\frac{\mu^{2}}{\gamma}+g(y))\geq0 \\ \mu(g(y)+\frac{1}{2}\gamma g(y)=\frac{1}{2\gamma}[\left\{-(\mu+\gamma g(y))\right\}^{2}-\mu^{2}], \\ \mathop{}\limits\mathop{}\limits\mathop{}\limits\mathop{}\limits\mathop{}\limits\mathop{}\limits\mathop{}\limits\mathop{}\limits\mathop{}\limits\mathop{}\limits\mathop{}\limits\mathop{}\limits\mathop{}\limits\mathop{}\limits\mathop{}\limits\mathop{}\limits\mathop{}\limits\mathop{}\limits\mathop{}\limits\mathop{}\limits\mathop{}\limits\mathop{}\limits\mathop{}\limits-(\frac{\mu^{2}}{\gamma}+g(y))<0 \end{matrix}\right.$ (16)

Substituting (14) and (16) into (13) gives:

$L(w,\mu,\lambda)=J(y)+J_{1}(y,\mu)+J_{2}(y,\lambda)$ (17)

where $J_{1}(y,\mu)=\frac{1}{2\gamma}[max\left\{0,\mu+\gamma g(y)-\mu^{2}\right\}]$corresponds to the inequality constraint, and $J_{2}(y,\lambda)=\lambda h(y)+\frac{1}{2}\gamma\left\|h(y)\right\|^{2}$corresponds to the equality constraint.

A Newton-like learning algorithm is used to find the optimal value as:

$\Delta w=-\eta\left(\frac{\partial^{2}L(w,\mu,\lambda)}{\partial w^{2}}\right)^{-1}\frac{\partial L(w,\mu,\lambda)}{\partial w}$ (18)

where $\eta$ is the learning rate. The Lagrange multipliers μ and $\lambda$ are updated as:

$\Delta\mu=max\{-\mu,\gamma g(y)\}$ (19)

$\Delta\lambda=\gamma h(y)$ (20)

Finally, this algorithm can be briefed as follows:

(1) Setting initial values of Lagrange multipliers $\mu$ and $\lambda$, and the update rate $\eta$.

(2) Whiten and decentralize all of the observations, normalize the reference to zero mean and unit variance.

(3) Setting an initial vector$w_{0}$, where $w_{0}\neq0$.

(4) Update $\mu$ and $\lambda$ by $\mu_{k+1}=\text{ }\mu_{k}+\Delta\mu$ and $\lambda_{k+1}=\text{ }\lambda_{k}+\Delta\lambda$.

(5) Update vector $w$ to $w_{k+1}=\text{ }w_{k}+\Delta w$ utilizing equation (18) , and normalize$w$ as $w=\text{ }w/\left\|w\right\|$.

(7) To minimize$\left|J(y)_{k+1}-J(y)_{k}\right|$ by $\left\|\Delta w\right\|$. If $\left\|\Delta w\right\|<\varepsilon$ (in this study $\varepsilon$=0.01), return to Step (4).

(8) Output the demixing vector $w$.

The algorithm in Matlab has been developed in a Single Trial extraction toolbox ([STEP^@1.0^](mailto:STEP@1.0)), to be freely downloaded in <http://www.chinaiom.org/v1/?page_id=2>.
